# Antibiotic stewardship implementation at the largest solid organ transplantation center in Asia: a retrospective cohort study

**DOI:** 10.1186/s12893-023-01991-y

**Published:** 2023-04-11

**Authors:** Mojtaba Shafiekhani, Mojtaba Shabani-Borujeni, Ava Karimian, Mohammad Javad MomeniTabar, Zahra Zare, Sara Arabsheybani, Afsaneh Vazin

**Affiliations:** 1 Transplant Research Center, Shiraz University of Medical Sciences, Shiraz, Iran; 2 Shiraz Transplant Center, Abu-Ali Sina Hospital, Shiraz University of Medical Sciences, Shiraz, Iran; 3 Department of Clinical Pharmacy, Faculty of Pharmacy, Shiraz University of Medical Sciences, Shiraz, Iran

**Keywords:** Solid organ transplantation, Antibiotic stewardship, Antibiotic resistance, Kidney transplantation, Liver transplantation

## Abstract

**Background:**

Using Antimicrobial stewardship programs (ASP) to monitor the use of antibiotics can lead to improved antibiotic use and reduced costs.

**Methods:**

This retrospective cohort study was done at Shiraz Organ Transplant Center, the largest transplant center in Asia. Antimicrobial use, cost, clinical outcomes, and antibiotic resistance pattern were evaluated before and after ASP.

**Results:**

This study included 2791 patients, 1154 of whom were related to the time before ASP and 1637 to the time after ASP. During the period of the research, a total of 4051 interventions were done. The use of all classes of antibiotics was significantly reduced by ASP, with 329 DDD/100PD before the intervention compared to 201 DDD/100PD after it (*p* = 0.04). In addition, the overall cost of antibiotics purchased was much lower after the ASP measures were implemented ($43.10 per PD) than before implementation of the ASP measures ($60.60 per PD) (*p* = 0.03). After the implementation of ASP, the number of MDR isolates was significantly reduced.

**Conclusion:**

The results of our study showed that the implementation of ASP significantly reduced the number and costs of antibiotics and also the number of resistant pathogens, but did not affect the patients’ length of stay.

**Supplementary Information:**

The online version contains supplementary material available at 10.1186/s12893-023-01991-y.

## Introduction

Solid-organ transplantation (SOT) is the best-known therapeutic option for numerous end-stage diseases in the acute and chronic stages. It has been associated with improved survival and enhanced quality of life for patients [1, 2]. In recent years, SOT has been associated with notable progress, and this improvement is more significant in the field of liver transplantation (LT) [3]. LT is currently considered the gold standard treatment for end-stage liver failure, with liver cirrhosis and hepatocellular carcinoma being the most common indications for LT worldwide [4]. Advances in surgical techniques, methods of diagnosis and prevention of infection, and immunosuppressive regimens have been associated with improvements in long-term post-transplant outcomes. However, surgical complications, infections, and rejection are some of the problems patients encounter after SOT [5]. Infection is the most common cause of death shortly after transplantation in many centers. Unfortunately, early detection of infection is delayed due to the effects of immunosuppressive agents, inhibition of normal inflammatory responses, and failure to recognize the signs associated with infection [6]. Infections caused by multidrug-resistant organisms (MDRs) have become more common in patients following (SOT). In these patients, prompt detection of infection and selection of suitable antibiotic treatment is linked to improved results [7]. End-stage patients who require organ transplants, such as dialysis or cirrhosis patients, have been exposed to broad-spectrum antibiotics prior to transplantation due to frequent hospitalizations, so the risk of developing microbial resistance is high [8]. Antimicrobial stewardship programs (ASPs) have received special support as the prevalence of antimicrobial resistance has increased. ASPs have improved antibiotic, antifungal, and antiviral therapies in clinical settings by promoting the selection of appropriate drug regimens, including dosing, duration of treatment, and route of administration [9–12]. However, there is a paucity of data documenting ASPs for SOT recipients, preventing us from fully comprehending their potential influence on this population [9, 13, 14]. As previously stated, the rise in SOT, along with an increase in antimicrobial resistance and a scarcity of new effective antimicrobial agents, necessitates the use of effective antibiotic therapy to improve the outcomes of the procedure [15]. Variables unique to the SOT population, such as the time after transplantation, intensity and duration of immunosuppression, type of organ transplanted, and donor-derived infections may be overlooked when stewardship methods are applied broadly. The dearth of clinical evidence on particular ASP interventions and successful treatment duration among SOT patients necessitates the development of customized ASP therapies [16]. In the present study, the clinical and economical outcomes of designing and implementing ASPs in SOT recipients for the first time at Shiraz Organ Transplant Center as the largest SOT center in Asia and worldwide are investigated.

## Methods

### Study setting

This retrospective single-center cohort study was conducted from March 2020 to November 2021 at Shiraz Organ Transplant Center, Shiraz, Iran which is affiliated to Shiraz University of Medical Sciences, the largest transplant center in Asia, which has 350 beds and performs over 600 solid organ transplants including the liver, kidney, pancreas-kidney, intestine, heart, and lungs, every year. At this center, all medical services are provided to End-Stage Renal Disease (ESRD) and end-stage liver and organ failure disease patients before and after transplantation. This study was approved by the Institutional Review Board and the Ethics Committee of Shiraz University of Medical Sciences (approval code: IR.SUMS.REC.1399.395). All of the protocols were based on the ethical guidelines of the 1975 Helsinki Declaration [17].

### Antibiotic Stewardship Program (ASP)

Before performing ASP (From March 2020 to December 2020 is related to the pre-ASP period) in the hospital, transplant surgeon specialists, gastroenterologists, or nephrologists prescribed antibiotics (empirical, directed, and surgical prophylaxis) based on clinical or laboratory evidence, and in cases of necessity, they consulted infectious disease specialists.

The ASP was established and implemented in the hospital in December 2020 (From January to November 2021, it is related to the Post ASP period). An infectious disease specialist, a clinical pharmacist, a skilled infectious disease nurse, a clinical microbiologist, and one of the organ transplant team surgeons were among the team members. Since 2020, annually, the resistance-susceptibility pattern of isolated pathogens of the hospital has been prepared separately for each ward and different classes of antibiotics by the antibiotic stewardship team have been the basis for the selection of antibiotics in each ward. Furthermore, based on the trials conducted [18], local guidelines for particular situations, such as prophylactic antibiotics for surgical site infection, have been prepared and provided to the transplant surgery team. The request for antibiotics is firstly sent to the hospital central pharmacy through the Hospital Information system (HIS) and is notified to the antibiotic stewardship team within 72 h; the team will evaluate the patient's prescribed antibiotic for the characteristics listed in Table 1 and, if necessary, change or modify any of them, while the treating physician is informed about the cases. All the services of this team were available seven days a week, 365 days a year. At the end of each month, ASP team members also informed the team members about the results of their interventions along with recommendations in a face-to-face or virtual meeting.

**Table 1:** Antibiotic characteristics evaluation in the antibiotic stewardship program among solid organ transplant recipients

**Section and Topics**	**Description**
Prescribing antibiotics	Proper Indications Start new antibiotics performing microbiological surveillance Possibility of Narrowing down antibiotic
Dose and interaction of antibiotics	Appropriate dose according to indications Dose Adjustment in renal & hepatic failure Drug-antibiotic interactions
Route of administration	Appropriate route Change route of administration from IV to PO
Duration of antibiotic administration	Intervals and frequency of administration Duration of therapy
Adhere to the ASP program	Filled necessary forms points of adherence

*IV* Intravenous, *PO* taken by mouth

### Data collection

All demographic, clinical, laboratory, and follow-up information of hospitalized patients during the whole study period was extracted from the hospital HIS system and electronic patient records. Also, all the interventions performed by the team were carefully recorded. The World Health Organization (WHO) Collaborating Center for Drug Statistics Methodology advised that antibiotic use should be standardized using the Anatomical Therapeutic Chemical (ATC) classification system and the DDD, as a measuring unit. The amount of antibiotics consumed by inpatients was measured in DDD/100 bed-days [19]. All the isolates from the patients' samples received from different wards to the central microbiology laboratory, including blood, wound swabs, sputum, drain fluids, and urine were collected to evaluate the sensitivity and resistance patterns. When conducting antimicrobial susceptibility testing and interpretation, we followed Clinical Laboratory Standard Institute (CLSI) criteria [20].

### Primary and secondary outcomes

The primary aim of the study was to compare antimicrobial use data expressed as DDD before and after the ASP. A secondary goal in our institute study included comparing the total cost of antibiotics as well as changes in antibiotic resistance patterns, with a focus on the (MDR) pathogens such as *Acinetobacter baumannii, Escherichia coli, Klebsiella pneumonia, Pseudomonas aeruginosa, Methicillin-resistant Staphylococcus aureus, and vancomycin-resistant enterococci* (VRE).

### Statistical analysis

SPSS software version 16 was used for all statistical analyses. The Smirnov-Kolmogorov test was used to determine whether the data were normally distributed, and statistical analysis was performed using t-test, Mann–Whitney test, or Wilcoxon test, depending on the distribution. *P* < 0.05 were considered significant.

## Results

**Fig. 1:**
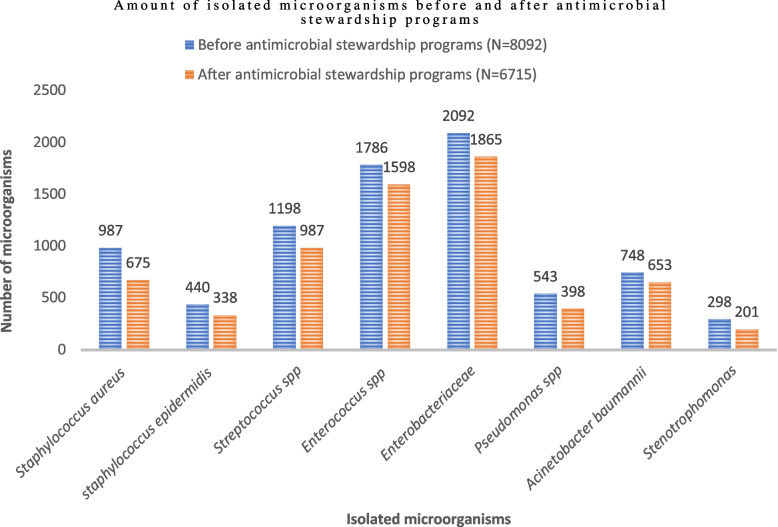
Amount of isolated microorganisms before and after antimicrobial stewardship programs (*N* = 14,807)

Two thousand seven hundred ninety-one patients participated in this study; they were waiting for transplantation or had undergone solid organ transplantation; of them, 1154 patients were related to the time before ASP and 1637 to the time after ASP. The mean age of this group of patients was 54.31 ± 13.21 years. The demographic information of the patients is shown in Table 2. The total number of 4051 interventions were made by ASP memberships during this study, as shown in Table 3. Measures performed by the ASP significantly reduced the use of all classes of antibiotics, so that before the intervention, 329 DDD/100PD, compared to 201 DDD/100PD (*p* = 0.04), was seen. Surgical wards have had a much higher reduction in the use of all classes of antibiotics than medical wards (*p* = 0.01). The clinical outcomes associated with ASP interventions are described in Table 4 and Additional file 1. Also, after the beginning of the ASP monitoring, the total cost of antibiotics purchased ($43.10 per PD) was significantly reduced compared to before ($60.60 per PD) the ASP measures (*p* = 0.03). The prevalence of different microorganisms before and after the ASP intervention is shown in Fig. 1.

**Table 2:** Demographic and clinical information of the SOT recipients in the Pre- and Post-ASP (*N* = 2791)

**Demographic information**	**Pre ASP intervention** ***N*** ** = 1154 (%)**	**Post ASP intervention** ***N*** ** = 1637 (%)**	***p*** **-value**
Age	55.01 ± 13.81	56.00 ± 14.01	0.77
Sex			
Male	671 (58.14%)	692 (42.27%)	0.52
Female	483 (41.85%)	945 (57.72%)	
Comorbidities			
DM	504 (43.67%)	609 (37.2%)	0.76
HTN	498 (43.15%)	557 (34.02%)	0.92
Cardiovascular disease	610 (52.85%)	595 (36.34%)	0.61
COPD or Asthma	102 (8.83%)	68 (4.15%)	0.70
DVT or PTE	41 (3.55%)	33 (2.01%)	0.14
ESRD	298 (25.82%)	434 (26.51%)	0.09
Liver cirrhosis	167 (14.47%)	491 (29.99%)	0.11
Transplantation status transplanted	689 (59.7%)	702 (42.88%)	0.08
Candidate for transplantation	465 (40.29%)	935 (57.11%)	
Type of SOT			
Liver	305 (44.26%)	339 (48.29%)	0.90
Kidney	352 (51.08%)	325 (64.29%)	0.07
MVT	9 (1.3%)	3 (0.42%)	0.10
SPK	14 (2.03%)	21 (2.99%)	0.85
Small bowel	5 (0.72%)	3 (0.42%)	0.13
Heart and lung	4 (0.58%)	11 (1.56%)	0.06
Time since transplantation(months)	53 ± 11.09	57.08 ± 10.98	0.48
Intensive care units’ admissions(n)	670	701	0.85

*DM* Diabetes mellitus, *HTN* Hypertension, *COPD* Chronic obstructive pulmonary disease, *DVT* Deep Vein Thrombosis, *PTE* pulmonary thromboembolism, *ESRD* End-Stage Renal Disease, *MVT* Multivisceral transplantation, *SPK* Simultaneous pancreas-kidney

**Table 3:** Antibiotic Stewardship team’s interventions among SOT recipients (*N* = 4051)

Type of interventions	Number
**Discontinue unnecessary antibiotics**	**2971**
Discontinue carbapenems	1001
Discontinue Metronidazole	871
Discontinue Aminoglycosides	508
Discontinue Cephalosporins	471
Discontinue Fluoroquinolones	88
Discontinue polymyxin E	26
Others	6
**Start necessary antibiotics**	**302**
Start carbapenems according microbiological surveillance	105
Start Antibiotic against Gram positive bacteria	101
Start beta-lactam beta-lactamase inhibitors according microbiological surveillance	96
**Deescalate broad spectrum antibiotics**	**221**
Switch carbapemens to beta-lactam beta-lactamase inhibitors	172
Switch linezolide to vancomycine	22
Switch vancomycine to anti-staphylococcal penicillins	16
Switch polymyxin E to Continuous high-dose infusion of carbapenems	11
**Optimized therapeutic dose of antibiotics**	**209**
Decrease total daily dose of antibiotics	177
Increase total daily dose dose of antibiotics	32
Adjust dose of antibiotics in renal impairment	217
Consider drug-antibiotics interactions	54
Change of IV route to PO form	77

**Table 4:** Clinical and financial outcomes of antibiotic stewardship interventions

**Clinical outcome**	**Pre-ASP intervention**	**Post-ASP intervention**	**Percentage-of changes (%)**	**Confidence interval (CI 95%)**	***p*** **-Value**
Confirmed infections(N)	1009	903	-10.50	(0.6–7.5)	**0.80**
**Amount of antibiotics used(N)**					
Third generation cephalosporins	14,569	12,431	-14.67	(1.1–3.7)	**< 0.001**
Fluoroquinolones	11,022	10,700	-2.92	(1.3–1.7)	**0.031**
Vancomycine	11,001	9920	-9.82	(2.3–6.9)	**< 0.001**
Carbapenems	10,980	10,542	-3.98	(0.9–1.4)	**0.089**
linezolide	4356	3009	-30.92	(1.8–3.3)	**0.043**
Beta-Lactam Beta-Lactamase Inhibitors	7892	5421	-31.31	(1.5–2.3)	**0.039**
Polymyxin E	6542	5040	-22.95	(1.2–2.4)	**0.01**
Aminoglycosie	7569	5090	-37.89	(0.8–1.6)	**0.081**
Metronidazole	12,341	10,070	-18.40	(0.6–1.8)	**0.066**
Frequency of patients with MRSA isolate (%)	77.20% (891/1154)	37.14% (608/1637)	-51.89	(1.3–2.9)	**0.03**
Frequency of patients with (%) CRE isolate	86.82% (1002/1154)	54.79% (897/1637)	-36.89	(1.9-3.3)	**0.01**
Frequency of patients with KPC isolate	68.45% 790/1549	24.49% 401/1637	-64.22	(2.5–7.5)	**< 0.001**
Frequency of patients with VRE colonization	55.54% (641/1154)	18.87% (309/1154)	-66.62	(3.3–6.4)	**0.029**
DDD/100 Patient Days	329	201	-38.91	(1.7–2.4)	**0.04**
Average Monthly Cost of antibiotics prescribed per patient (USD)	137	104	-24.09	(1.3–2.6)	**0.03**
Total cost of antibiotics (USD)	19,956	15,498	-22.33	(2.2–5.3)	**< 0.001**
Mean duration of antibiotic use (days) (Mean ± SD)	26.11 ± 11.19	21.90 ± 9.00	-16.12	(1.2–3.4)	**0.04**
Length of hospital stay (Days)	38.01 ± 17.14	32.00 ± 12.01	-15.81	(0.8–2.1)	**0.98**
Length of ICU stay (Days)	14.29 ± 4.91	12.11 ± 5.60	-15.25	(0.7**–**2.1)	**0.77**
Mortality due to infectious causes	202	198	-1.98	(0.6–2.2)	**0.08**

*MRSA* Methicillin-resistant Staphylococcus aureus, *CRE* carbapenem-resistant Enterobacterales, *KPC* Klebsiella pneumoniae carbapenemase, *VRE* Vancomycin-resistant Enterococci, *USD* U.S. dollar, CI; confidence interval

In comparison between the patients waiting for transplantation and transplanted ones, the results of our study showed that the percentage of reduction in the usage of third generation cephalosporins (-13.90% vs. -18.92%), carbapenems.

(-2.00% vs. -4.01%) and linezolid (-28.80% vs. -32.00%) in transplant patients was significantly lower than those waiting for transplant after ASP implementation (*p* < 0.001).

Also, 51.59% of the ASP team interventions were for patients awaiting transplant, so that the most interventions in this category of patients included discontinuing unnecessary antibiotics, optimizing the dosage, and adjusting the antibiotic dosage based on GFR. Meanwhile, the most interventions of the ASP team among post-transplant patients included de-escalating broad spectrum antibiotic, starting necessary antibiotics, and discontinuing unnecessary antibiotics, respectively.

The mean duration of antibiotic use in patients candidate for transplantation is significantly longer than the transplanted ones before performing ASP (27.12 ± 13.10 vs. 20.18 ± 12.00 days, *p* = 0.021); after performing ASP, the mean duration of antibiotic use in patients waiting for transplant was significantly decreased compared to the transplanted patients (11.40 ± 25 vs. 11.90 ± 18.00, *p* = 0.03).

## Discussion

In our study, the number and costs of antibiotics and also the number of resistant pathogens were significantly lower following the ASP implementation and monitoring. Our results demonstrated the highest reduction in consumption with linezolid, beta-lactam/beta-lactamase inhibitors, and aminoglycosides. Reducing the usage of these antibiotics plays an important role in reducing MDR. The most common ASP program interventions in our study was the discontinuation of unnecessary antibiotics, most of which were aminoglycosides, beta-lactams beta-lactamase inhibitors, and linezolid. Many studies on ASP have also concluded that most antibiotics are unnecessary in most cases. According to the research conducted by Cusini et al., 32% of all antibiotics were given unnecessarily; it occured more in the surgical wards than in the medical wards [21]. Impaired inflammatory response due to suppression of the immune system reduces the signs and symptoms of an aggressive infection; therefore, it is essential to start taking antibiotics as soon as possible before the infection has spread [22]. In patients with immunocompromised status, discontinuation or de-escalation of broad-spectrum antibiotics should be done carefully as needed. As noted by Wooyoung Jang et al., inappropriate de-escalation of broad-spectrum antibiotics can result in a significant increase in the use of other classes of antibiotics, in addition to causing treatment failure [23]. Although this trend was not observed in our study, given that the duration of follow-up was one year, this issue should be examined in a larger period of time. These results also indicate the need to form an ASP team and carefully discontinue or de-escalate the antibiotics based on the patient's clinical condition. According to numerous studies on post-transplant infections, MDR pathogens, particularly gram-negative bacteria, are found in the majority of post-transplant infections [8, 24]. Most patients are colonized with a wide spectrum of resistant pathogens as a result of their long-term hospitalization before and after transplantation [25]. Over the course of a year, Cheon et al. found that strict antimicrobial stewardship, especially for carbapenems, significantly reduced the endemic MDR A. baumannii in the intensive care unit [26]. As a result, the ANTARCTICA coalition (Antimicrobial Resistance in Critical Care) has identified the implementation of ASP as one of the top priorities for preventing resistant bacterial colonization in hospitals [27]. ASPs can offset or reduce the costs while enhancing some patient outcomes, implying that they are of great value to some healthcare systems [28]. According to the IDSA/SHEA standards, comprehensive prevention programs can reduce antimicrobial use by 22–36 percent, resulting in significant cost savings [29]. The results of our study showed that the implementation of ASP significantly reduced the duration of antibiotic administration and consequently reduced the monthly and total cost of antibiotics. According to the results of our study, implementation of ASP significantly reduced the duration of antibiotic administration, and thus the monthly and total cost of antibiotics. The most significant cost savings were in the categories of carbapenems, beta-lactam/beta-lactamase inhibitors, and polymyxin, which saved a total of 190,000 dollars in a year by reducing antibiotic use. According to the results of a systematic review study [30], the ASP implementation resulted in a reduction of $448.25 per 100 patient days. Although the patients' length of stay was reduced after ASP implementation, the difference was not statistically significant. In this regard, some studies have found that implementing ASP has resulted in a significant reduction in the length of stay [30]. However, some studies have found no effect on patient duration of stay, and in some cases the length of stay has even risen after ASP [31]. It is important to remember that several potential confounders affect the entire hospital stay, making it difficult to correctly assess the impact of ASP. Infection-related hospitalization should better reflect the impact of an ASP and should be considered an important endpoint in future studies.

In addition to demonstrating the benefits of ASP interventions, such as increased compliance with the guidelines, reduced costs, and lower resistance rates, the results of our study should be considered with caution as it has a number of limitations. For example, only antibacterial drugs were evaluated in this study, but because antifungal and antiviral drugs are also utilized in patients undergoing SOT, ASP programs for these drugs should be considered as well. Also, a detailed and comprehensive analysis of time series cannot be done from retrospective studies, and this requires prospective studies.

Furthermore, our study lasted a year, whereas more accurate data can be extracted in greater detail over a longer length of time. The pattern of consumption resistance has been studied based on the phenotype, and due to the limited cost of the project, it has not been possible to study the genotypes of antibiotic resistance.

## Conclusions

In conclusion, our study showed that, the number and costs of antibiotics and also the number of resistant pathogens were significantly lower following the ASP implementation and monitoring.

## Supplementary Information

**Additional file 1.**

## Data Availability

The datasets generated and/or analyzed during the current study are not publicly available since they contain information that could compromise the privacy of research participants but are available from the corresponding author on reasonable request.

## Abbreviations

ASP: Antimicrobial stewardship programs
SOT: Solid-organ transplantation
ESRD: End-Stage Renal Disease
HIS: Hospital Information system
IV: Intravenous
PO: Taken by mouth
ATC: Anatomical Therapeutic Chemical
CLSI: Clinical Laboratory Standard Institute
MDR: Multiple drug resistance
VRE: Vancomycin-resistant enterococci
WHO: World Health Organization
DM: Diabetes mellitus
HTN: Hypertension
COPD: Chronic obstructive pulmonary disease
DVT: Deep Vein Thrombosis
PTE: Pulmonary thromboembolism
MVT: Multivisceral transplantation
SPK: Simultaneous pancreas-kidney

## References

[1] Silva J, Fernández-Ruiz M, Aguado J (2018). Multidrug-resistant Gram-negative infection in solid organ transplant recipients: implications for outcome and treatment. Curr Opin Infect Dis.

[2] Kritikos A, Manuel O (2016). Bloodstream infections after solid-organ transplantation. Virulence.

[3] Jadlowiec CC, Taner T (2016). Liver transplantation: current status and challenges. World J Gastroenterol.

[4] Finotti M, Auricchio P, Vitale A (2021). Liver transplantation for rare liver diseases and rare indications for liver transplant. Transl Gastroenterol Hepatol.

[5] Idossa DW, Simonetto DA (2017). Infectious complications and malignancies arising after liver transplantation. Anesthesiol Clin.

[6] Moon DB, Lee SG (2009). Liver transplantation. Gut Liver..

[7] Hand J (2018). Strategies for antimicrobial stewardship in solid organ transplant recipients. Infect Dis Clin.

[8] Bartoletti M, Giannella M, Tedeschi S (2018). Multidrug-resistant bacterial infections in solid organ transplant candidates and recipients. Infect Dis Clin.

[9] Barlam TF, Cosgrove SE, Abbo LM (2016). Implementing an antibiotic stewardship program: guidelines by the Infectious Diseases Society of America and the Society for Healthcare Epidemiology of America. Clin Infect Dis.

[10] Holmes AH, Moore LSP, Sundsfjord A (2016). Understanding the mechanisms and drivers of antimicrobial resistance. Lancet.

[11] USA. Report to the President on Combating Antibiotic Re; Executive Office of the President. President’s Cou; September 2014. 2014;(September).

[12] Fishman N, America S for HE of, America IDS of (2012). Policy statement on antimicrobial stewardship by the society for healthcare epidemiology of America (SHEA), the infectious diseases society of America (IDSA), and the pediatric infectious diseases society (PIDS). Infect Control Hosp Epidemiol.

[13] Baur D, Gladstone BP, Burkert F (2017). Effect of antibiotic stewardship on the incidence of infection and colonisation with antibiotic-resistant bacteria and Clostridium difficile infection: a systematic review and meta-analysis. Lancet Infect Dis.

[14] So M, Yang DY, Bell C (2016). Solid organ transplant patients: are there opportunities for antimicrobial stewardship?. Clin Transplant.

[15] So M, Morris AM, Nelson S (2019). Antimicrobial stewardship by academic detailing improves antimicrobial prescribing in solid organ transplant patients. Eur J Clin Microbiol Infect Dis.

[16] So M, Hand J, Forrest G, et al. White paper on antimicrobial stewardship in solid organ transplant recipients. Am J Transplant. 2022;22(1):96–112. 10.1111/ajt.16743 PMC9695237 34212491

[17] Association WM (2001). World Medical Association Declaration of Helsinki. Ethical principles for medical research involving human subjects. Bull World Health Organ.

[18] Shafiekhani M, Karimzadeh I, Nikeghbalian S (2020). Comparison of Ceftizoxime Plus Ampicillin-Sulbactam versus Gentamicin Plus Ampicillin-Sulbactam in the Prevention of Post-Transplant Early Bacterial Infections in Liver Transplant Recipients: A Randomized Controlled Trial. Infect Drug Resist..

[19] Rønning M (2009). Coding and classification in drug statistics – From national to global application. Nor Epidemiol.

[20] Performance C (2016). Standards for Antimicrobial Susceptibility Testing, CLSI Supplement M100S.

[21] Cusini A, Rampini SK, Bansal V (2010). Different patterns of inappropriate antimicrobial use in surgical and medical units at a tertiary care hospital in Switzerland: a prevalence survey. PLoS One.

[22] Fishman JA, Rubin RH (1998). Infection in organ-transplant recipients. N Engl J Med.

[23] Jang W, Hwang H, Jo H (2021). Effect of discontinuation of an antimicrobial stewardship programme on the antibiotic usage pattern. Clin Microbiol Infect.

[24] Shafiekhani M, Mirjalili M, Vazin A (2019). Prevalence, risk factors and treatment of the most common gram-negative bacterial infections in liver transplant recipients: a review. Infect Drug Resist.

[25] Nguyen MH, Shields RK, Chen L (2022). Molecular epidemiology, natural history and long-term outcomes of multi-drug resistant Enterobacterales colonization and infections among solid organ transplant recipients. Clin Infect Dis..

[26] Cheon S, Kim M-J, Yun S-J (2016). Controlling endemic multidrug-resistant Acinetobacter baumannii in intensive care units using antimicrobial stewardship and infection control. Korean J Intern Med.

[27] De Waele JJ, Akova M, Antonelli M (2018). Antimicrobial resistance and antibiotic stewardship programs in the ICU: insistence and persistence in the fight against resistance. A position statement from ESICM/ESCMID/WAAAR round table on multi-drug resistance. Intensive Care Med.

[28] Nathwani D, Varghese D, Stephens J (2019). Value of hospital antimicrobial stewardship programs [ASPs]: a systematic review. Antimicrob Resist Infect Control.

[29] Drew RH (2009). Antimicrobial stewardship programs: how to start and steer a successful program. J Manag Care Pharm.

[30] Huebner C, Flessa S, Huebner NO (2019). The economic impact of antimicrobial stewardship programmes in hospitals: a systematic literature review. J Hosp Infect.

[31] Palmay L, Elligsen M, Walker SAN (2014). Hospital-wide rollout of antimicrobial stewardship: a stepped-wedge randomized trial. Clin Infect Dis.

