# Supplementary material for: Antibiotic stewardship implementation at the largest solid organ transplantation center in Asia: a retrospective cohort study
Source: BMC Surg. 2023 Apr 11;23:81. doi: 10.1186/s12893-023-01991-y (PMC10091536; doi:10.1186/s12893-023-01991-y)
Supplement: Supplementary file 1 — Additional file 1. [file 12893_2023_1991_MOESM1_ESM.docx]

**Additional file 1** mean duration of antibiotic use and total cost before and after antibiotic stewardship implementation

| **mean duration of antibiotic use and total cost** | **Pre-ASP intervention** | **Post-ASP intervention** |
| --- | --- | --- |
| 3th generation cephalosporin  Mean±SD duration time  Total cost (USD) | 9.51±2.20  13124 | 7.00±2.19  11100 |
| Fluoroquinolones  Mean ±SD duration time  Total cost (USD) | 16.00±9.87  17200 | 13.21±7.80  15590 |
| Vancomycine  Mean ± SD duration time  Total cost (USD) | 15.10±3.33  19129 | 13.22±8.00  16200 |
| Carbapenems  Mean± SD duration time  Total cost (USD) | 17.68±11.10  21340 | 15.38±7.71  18500 |
| linezolide  Mean ±SD duration time  Total cost (USD) | 15.32±10.0  18760 | 14.11±6.32  16339 |
| Beta-lactam/beta-lactamase inhibitors  Mean±SD duration time  Total cost (USD) | 18.11±12.01  20210 | 15.22±10.02  18651 |
| Polymyxin  Mean ±SD duration time  Total cost (USD) | 20.17±13.11  18230 | 18.00±7.71  16320 |
| Aminoglycoside  Mean ±SD duration time  Total cost (USD) | 15.20±7.22  13900 | 13.30±5.69  1224 |
| Metronidazole  Mean ±SD duration time  Total cost (USD) | 12.86±4.00  13200 | 11.19±4.22  11490 |
